# The Physico-Chemical Properties of Dietary Fibre Determine Metabolic Responses, Short-Chain Fatty Acid Profiles and Gut Microbiota Composition in Rats Fed Low- and High-Fat Diets

**DOI:** 10.1371/journal.pone.0127252

**Published:** 2015-05-14

**Authors:** Frida Fåk, Greta Jakobsdottir, Evelina Kulcinskaja, Nittaya Marungruang, Chrysoula Matziouridou, Ulf Nilsson, Henrik Stålbrand, Margareta Nyman

**Affiliations:** 1 Food for Health Science Centre, Lund University, Lund, Sweden; 2 Department of Biochemistry and Structural Biology, Lund University, Lund, Sweden; National Institute of Agronomic Research, FRANCE

## Abstract

The aim of this study was to investigate how physico-chemical properties of two dietary fibres, guar gum and pectin, affected weight gain, adiposity, lipid metabolism, short-chain fatty acid (SCFA) profiles and the gut microbiota in male Wistar rats fed either low- or high-fat diets for three weeks. Both pectin and guar gum reduced weight gain, adiposity, liver fat and blood glucose levels in rats fed a high-fat diet. Methoxylation degree of pectin (low, LM and high (HM)) and viscosity of guar gum (low, medium or high) resulted in different effects in the rats, where total blood and caecal amounts of SCFA were increased with guar gum (all viscosities) and with high methoxylated (HM) pectin. However, only guar gum with medium and high viscosity increased the levels of butyric acid in caecum and blood. Both pectin and guar gum reduced cholesterol, liver steatosis and blood glucose levels, but to varying extent depending on the degree of methoxylation and viscosity of the fibres. The medium viscosity guar gum was the most effective preparation for prevention of diet-induced hyperlipidaemia and liver steatosis. Caecal abundance of *Akkermansia* was increased with high-fat feeding and with HM pectin and guar gum of all viscosities tested. Moreover, guar gum had distinct bifidogenic effects independent of viscosity, increasing the caecal abundance of *Bifidobacterium* ten-fold. In conclusion, by tailoring the viscosity and possibly also the degree of methoxylation of dietary fibre, metabolic effects may be optimized, through a targeted modulation of the gut microbiota and its metabolites.

## Introduction

Dietary fibres are degraded by the gut microbiota in the colon, giving rise to short-chain fatty acids (SCFA) that can be utilized by colonocytes as energy, absorbed into the systemic circulation or excreted in faeces. It has been established that ingestion of dietary fibres can improve the metabolic profile in humans and supplementation of SCFA to high-fat diet-fed rodents can reduce weight gain [1]. Interestingly, the effect on weight gain by SCFA can be ascribed not only to suppression of feed intake, but also through changes in energy expenditure [1, 2]. However, orally supplemented SCFA might affect metabolism through other mechanisms than SCFA formed during colonic fermentation of dietary fibres.

A high intake of dietary fat can induce low-grade systemic inflammation as well as insulin resistance in animal models [3], both factors are key aspects of the metabolic syndrome. Efficacy of dietary fibres in reduction of diet-induced obesity has been demonstrated in both animal models and in clinical trials. For instance, soluble fibre administration in the form of apple pectin, cocoa fibre or β-glucan was able to reduce inflammatory markers and oxidative stress in Zucker rats fed a high-fat diet [4]. A recent study evaluated effects of two highly viscous fibres (hydroxypropyl methylcellulose (HPMC) and guar gum) on adiposity and liver steatosis in high-fat-fed rats and found that both fibres reduced weight gain, fat mass and liver steatosis [5]. The authors emphasized that both the non-fermentable HPMC fibre and the fermentable guar gum reduced adiposity and hepatic steatosis [5], illustrating the importance of viscosity, and not the degree of fermentation, for metabolic effects of fibres.

The gut microbiota is highly influenced by the host diet and the fermentability of ingested fibres. Culture-independent, DNA-based techniques of identifying alterations in gut microbiota composition have provided new insight into the effect of fibres and these findings need to be evaluated in combination with other biomarkers. A recent *in vitro* study comparing the microbiota in faecal samples after fermentation of different fibres showed specific changes related to each investigated fibre [6]. Pectin induced increases in *Actinobacteria* (more specifically bifidobacteria), whereas guar gum samples had increased levels of *Bacteroidetes* and reductions in *Firmicutes* and *Proteobacteria* [6]. Similarly, we previously observed increases in the genus *Bacteroides* in rats fed guar gum [7]. Another study with apple pectin showed similar results; intake of apples in eight humans resulted in increased faecal numbers of *Bifidobacterium* and concurrent decrease in *Bacteroides* [8]. Guar gum galactomannan is known to be more or less completely fermented in the human colon [9]. However, the degradation mechanism of galactomannan by the gut microbiota is largely unknown. When human subjects were fed partially hydrolysed guar gum (Sunfibre, 20 kDa), *Bifidobacterium* spp. increased, while *Bacteroides* spp. did not [10]. The number of *Akkermansia muciniphila* was increased in mice fed with guar gum compared to mice that were fed other dietary fibres [11].

The physical and chemical properties of fibres have been shown to influence the impact of fibres on physiological parameters. A recent study compared the effects of varying viscosities and fermentability of fibres on adiposity and satiety hormones in rats and found that there was a complex interaction between viscosity and fermentation and that both properties played a role in food intake [12]. Another study on pectins with different degrees of methoxylation and viscosity emphasized the importance of both these parameters as well as molecular weight on cholesterol-lowering properties in humans [13]. A high viscosity fibre blend reduced LDL cholesterol more than a low viscosity wheat bran [14]. However, the impact of different fibre preparations on SCFA profiles has not been thoroughly investigated. As SCFA play a key role in effects on adiposity and metabolism [15], the SCFA pattern formed after ingestion of different fibre preparations warrants further investigation. Moreover, how viscosity and methoxylation of fibres affect the gut microbiota composition has, to our knowledge, not been examined. In the present study, we compared two pure preparations of dietary fibres, guar gum (a galactomannan) and pectin (a rhamnogalactouronan) with different physico-chemical properties, on weight gain, SCFA profiles, cholesterol and glucose levels and liver steatosis in high-fat fed rats. Moreover, we quantified the levels of three bacterial genera known previously to be affected by fibres: *Lactobacillus*, *Bifidobacterium*, and *Bacteroides*, as well as the levels of the species *Akkermansia muciniphila*, in caecal samples. We found that both the methoxylation and the viscosity of the fibres shaped the responses in the rats. The effects were linked to altered SCFA profiles and specific changes in the gut microbiota.

## Materials and Methods

### Animals

Male Wistar rats were divided into 12 groups of 7 animals. They were allowed to adapt to the environment for 6 days and thereafter the 3 weeks long experimental period followed. Five different dietary fibre preparations were fed to the rats and wheat starch served as a fibre-free control (Table 1). The wheat starch used contained no indigestible components (resistant starch) and did not give rise to any SCFA. Both the dietary fibre diets and the fibre-free diet contained either low (50 g/kg fat (maize oil) or high fat (250 g/kg lard and cholesterol, 50 g/kg maize oil) content. Each rat was given 12 g/day of food during the first two weeks of the experiment, followed by 20 g/day in the final week. The rats consumed the entire amount of administered feed. Water was given *ad libitum*. The rats were healthy and active throughout the experiment and the diets were well tolerated. However, some rats in the low-methoxylated (LM) pectin group initially lost weight during the study, which might indicate that this preparation of pectin was less well tolerated by the rats. At the end of the experiment, the rats were anaesthetized with a subcutaneous injection of a mixture (1:1:2) of Hypnorm (Division of Janssen-Cilage Ltd, Janssen Pharmaceutica, Beerse, Belgium), Dormicum (F. Hoffman-La Roche AG, Basel, Switzerland) and sterile water, at a dose of 0.15 ml/100 g body weight. The study was carried out in accordance with the Lund University ethical guidelines and recommendations regarding animal experiments. The Malmö/Lund Ethical Committee on Animal Research, Sweden approved the study (ethical approval number M56-12). All efforts were made to minimize suffering of the rats.

**Table 1:** Fibre processing and composition (g/kg, dwb) of experimental diets.

Diet	Fibre-free	Guar-gum	Pectin
Processing	None	Low, Medium or High viscosity	Low (24%) or High (70%) Methoxylation
Dietary fibre	-	80	80
Lard	Low-fat diet—	Low-fat diet—	Low-fat diet –
Lard	High-fat diet: 230	High-fat diet: 230	High-fat diet: 230
Casein	200	200	200
Rapeseed oil	50	50	50
Sucrose	100	100	100
Wheat starch*	Low-fat diet: 591	Low-fat diet: 491–503	Low-fat diet: 491–503
Wheat starch*	High-fat diet: 341	High-fat diet: 241–253	High-fat diet: 241–253
Cholesterol	Low-fat diet—	Low-fat diet—	Low-fat diet—
Cholesterol	High-fat diet: 20	High-fat diet: 20	High-fat diet: 20
DL-Methionine	1.2	1.2	1.2
Choline chloride	2	2	2
Mineral mixture	48	48	48
Vitamin mixture	8	8	8

* Varied according to the dietary fibre content of the test materials.

### Diets

Pectin extracted from citrus peels (Danisco, Copenhagen, Denmark) with two different degrees of methoxylation (approximately 24%, low methoxylation (LM) and high methoxylation (HM) 70%) and guar gum with low, medium or high viscosity were used in the study (Table 1). The high viscosity guar gum (Grindsted Guar 250) and the medium viscosity guar gum (Meyprodor 50) were obtained from Danisco, Norrköping, Sweden. Meyprodor 50 is a type of partially hydrolysed guar gum and this material was used to produce low viscosity guar gum.

### Preparation of low viscosity guar gum

Partially hydrolysed guar gum (Meyprodor 50, Danisco, Norrköping, Sweden) was mixed at a concentration of 22 g/l with 10 mM Na—citrate buffer pH 5.0 in a kitchen blender and incubated overnight at 4°C. Hydrolysis within the galactomannan backbone was then accomplished by adding *Aspergillus niger* β-mannanase (Megazyme, Bray, Ireland) [16] to a concentration of 16 nanokatal (nkat) per gram of guar gum (360 nkat/l hydrolysis mixture [17]) and incubated at 40°C for 9 h with occasional stirring. After adjusting pH to 9.5 with NaOH the hydrolysis mixture was heated to 80°C and incubated at that temperature for 45 min to denature the enzyme. When the mixture had been cooled to room temperature, the pH was adjusted to 7.6 by adding HCl. The hydrolysed guar gum was then freeze dried.

### Analyses on raw materials

#### Viscosity measurements of guar gum

The freeze-dried preparations were re-dissolved in water, giving a final concentration of 2% w/w. The viscosity was measured by a Malvern Instrument (Malvern Instruments Ltd, Malvern, United Kingdom).

#### Relative fluidity measurement

1% (w/v) solution of each guar gum preparation was used in the analysis. The time the materials travelled 10 cm on a consistometer (Christison Particle Technologies, UK) was measured in triplicate to generate relative fluidity data of the different types of guar gum.

#### Oligo- and monosaccharide analysis of guar gum

Since the enzymes may degrade the guar gum extensively the content of mono- and oligosaccharides was quantified. The guar gum preparations were dissolved in MilliQ water, filtered through a 0.2 μm filter and analysed by high performance anion exchange chromatography with pulsed amperometric detection (HPAEC-PAD) (Dionex, Sunnyvale, CA) [18]. For oligosaccharide separation, CarboPac PA-100 column was used with an isocratic flow of 78 mM NaOH. Manno-oligosaccharides were used as standards. CarboPac PA-10 column with an isocratic flow of 1 mM NaOH 1 ml/min was used for monosaccharide analysis. Oligosaccharide content was estimated by use of the peak areas of mannotriose and mannopentaose standards.

#### Size exclusion chromatography of guar gum

Size exclusion chromatography (SEC) was used to estimate average molecular weight, M_w_, of the low viscosity guar gum similar to previously described [19]. SEC was run on an FPLC system (Pharmacia Biotech, Uppsala, Sweden) using Superdex 75 10/300 and Superdex 200 10/300 columns (Pharmacia Biotech, Uppsala, Sweden) in parallel coupled to a refractive index detector (Erma-inc, Tokyo, Japan) [20]. MilliQ water was used as mobile phase at a flow of 0.3 ml/min. The guar gum was diluted to 1 g/l in water, centrifuged at 10 000 g for 20 min and then filtered through a 0.22 μm filter and injected using a 500 μl loop. Dextrans (Fluka, Buchs, Switzerland) with M_w_ of 1270, 5220, 80900, 273 000 and 409800 Da were used as molecular weight standards.

### Analyses on rats

#### SCFA in portal blood and caecal content

SCFA in serum samples and caecal content were analysed as described previously [7]. GC ChemStation software (Agilent Technologies Inc., Wilmington, DE, USA) was used to analyse the data.

#### Liver fat, cholesterol and triglycerides

Liver fat, liver cholesterol and triglyceride (TG) levels, as well as blood cholesterol and TG levels were quantified as described in previous work [7].

#### Blood glucose

Peripheral blood samples were analysed at time of dissection using a Hemocue Glucose 201 System (Hemocue, Ängelholm, Sweden). Rats were administered the final feed the morning before the day of dissection and were thus fasted over-night before blood sampling.

#### Caecal microbiota

DNA from caecal content was extracted using the QIAamp DNA Stool Kit (Qiagen, Hilden, Germany) according to manufacturer’s instructions with an addition of glass beads beating and a lysis temperature of 95°C to increase disruption of bacterial cell walls. 16s rRNA genes of bacteria belonging to *Bifidobacterium*, *Lactobacillus* and *Bacteroides* genera and the species *Akkermansia muciniphila* were quantified using qPCR with specific primer sets (Table 2). PCRs were performed with 20 μL final volume in 96-well PCR plates. Each reaction contained 10 μL SsoAdvanced Universal SYBR Green Supermix (Bio-rad, USA), 0.3 μM of each primer, 2 μL of template DNA, and RNase-free water to the final volume of 20 μL. The thermal cycling was performed on a CFX96 Touch Real-Time PCR Detection System (Bio-rad, USA). The amplification program comprised of 95°C for 3 min, followed by 40 cycles of denaturation at 95°C for 10 s, annealing and elongation at 60°C for 20 s for *Lactobacillus* and *Bacteroides*, and for 30 s for *Akkermansia muciniphila* and *Bifidobacterium*. Melt curve analysis was performed immediately after amplification, to ensure primer specificity. Absolute quantification of unknown samples was calculated based on standard curves generated by CFX manager software (Bio-rad, USA), with dilution series of known concentrations of plasmid DNA containing cloned target sequences for each set of primers. Amount of bacteria was expressed as copy numbers of 16S rRNA genes per gram of caecal content.

**Table 2:** Sequences of PCR primers.

Target bacteria	Sequence (5’-3’)	Amplicon size (bp)	Reference
*Akkermansia muciniphila*	Akk-F: CAGCACGTGAAGGTGGGGAC	327	[21]
	Akk-R: CCTTGCGGTTGGCTTCAGAT		
*Bifidobacterium*	Bifido-F: TCG CGT CYG GTG TGA AAG	243	[22]
	Bifido-R: CCA CAT CCA GCR TCC AC		
*Lactobacillus*	Lacto-F: GGAATCTTCCACAATGGACG	217	[23]
	Lacto-R: CGCTTTACGCCCAATAAATCCGG		
*Bacteroides*	Bact-F: GAGAGGAAGGTCCCCCAC	106	[24]
	Bact-R: CGCTACTTGGCTGGTTCAG		

### Statistical analysis

One-way ANOVA with Dunnett’s multiple comparison test was used to analyse significant differences (*P*<0.05) between the fibre-free control groups (low- or high-fat diet) and corresponding fibre groups. Values are reported as mean and standard deviation, unless otherwise noted. Statistical calculations were performed using Graphpad Prism 6, version 6.04 (Graphpad Software Inc, La Jolla, USA). For generation of the PCA plot, SIMCA software was used (Umetrics AB, Umeå, Sweden). Minitab 16 Statistical Software (Minitab Ltd, Coventry, United Kingdom) was used to perform two-way ANOVA to test interactions and main effects of each factor (fat, fibre) on the caecal microbiota.

## Results

### Physico-chemical characterization of guar gum

The monosaccharide analysis of low viscosity guar gum revealed traces of monosaccharides (0.01 and 0.25% galactose and glucose, respectively (w/w)) (Table 3). The medium viscosity guar gum contained minor amounts of glucose (0.13% w/w). The low and medium viscosity guar gum contained less than 1.5% w/w oligosaccharides. The high viscosity guar gum was estimated to contain 0.11% oligosaccharides and no monosaccharides were detected (Table 3).

**Table 3:** Physico-chemical properties of guar gum.

	Guar gum low viscosity	Guar gum medium viscosity	Guar gum high viscosity
M_w_, kDa	315	900^1^	2000–3000^1^
Relative fluidity, s	1	9	237
Oligosaccharides^2^	1.4	1.4	0.11
Monosaccharides^2^	0.26	0.26	ND

^1^data from manufacturer

^2^ w/w %

ND = not detected

The M_w_ of the low viscosity guar gum was estimated to be 315 kDa, shown in Table 3 together with the Mw of the high (2000–3000 kDa) and medium (900 kDa) viscosity guar gum.

**Fig 1:**
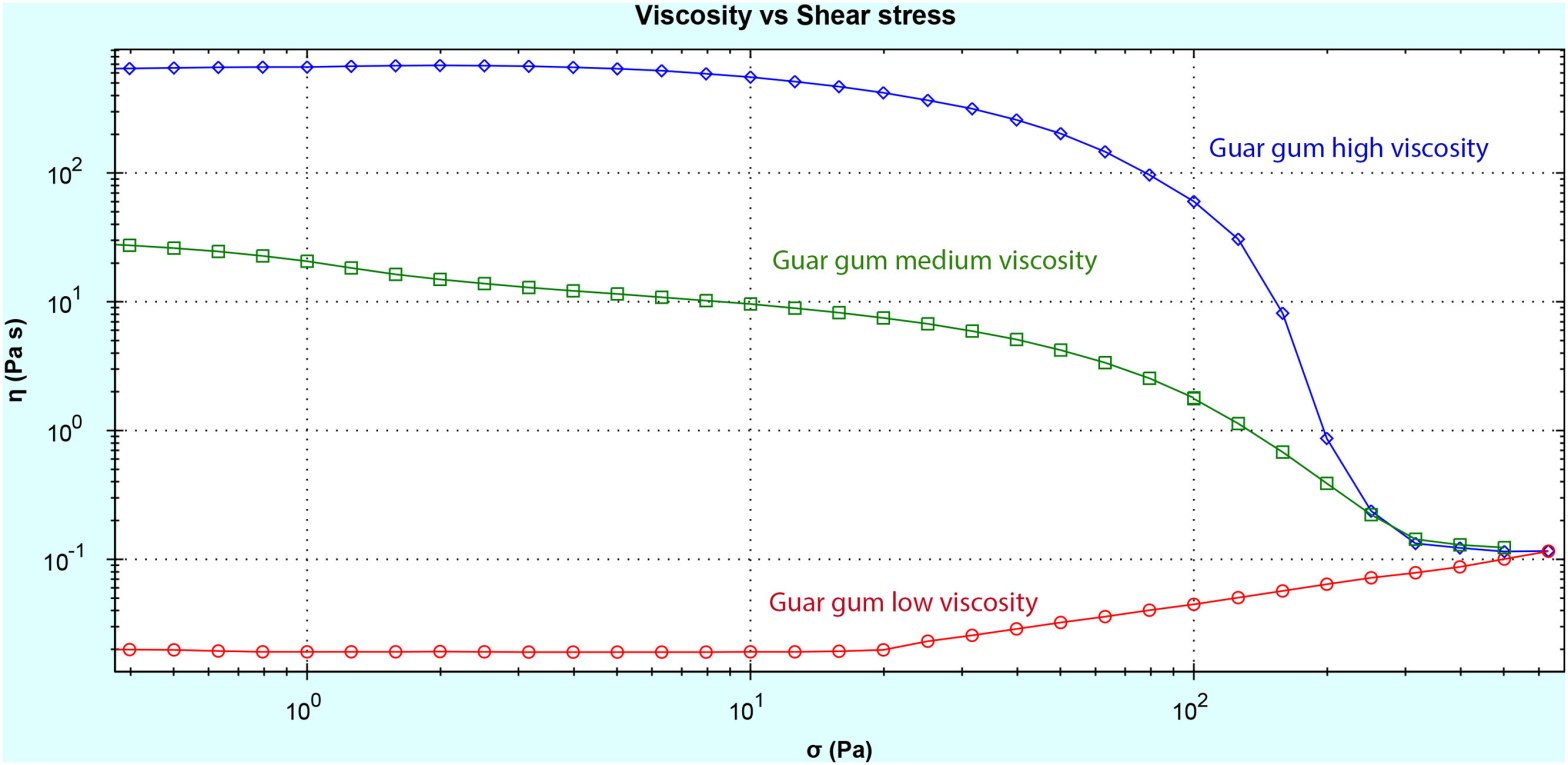
Viscosity vs. shear stress of guar gum with low, medium and high viscosity.

The viscosity of the different types of guar gum was highly dependent on the molecular weight, as expected, and guar gum with the highest molecular weight had the highest viscosity and vice versa (Fig 1). All materials showed a pseudo-plastic behaviour, i.e. the viscosity decreased with increasing shear stress, with the exception of low viscosity guar gum, which displayed increased viscosity with increasing shear stress. At shear stress 0.3 Pa (the lowest shear rate analysed and most comparable to the low shear rate in stomach) the viscosity was 600, 30 and 0.02 Pa ^●^ s, for high, medium and low viscosity guar gum, respectively. The relative fluidity measurement showed that the high viscosity guar gum needed 237 s to travel 10 cm, while medium and low viscosity required 9 and 1 s, respectively (Table 3).

### Diet-induced obesity

Initial starting weights did not differ significantly between groups, while at the end of the experiment, the high-fat diet fed group weighed significantly more than the low-fat diet fed group (*P* = 0.012, Table 4, S1). In general, the addition of either pectin with different degrees of methoxylation or guar gum with different viscosities, respectively, resulted in decreased weight gain as compared to the fibre-free control group. More specifically, in the low-fat diet-fed groups, the LM pectin group displayed lower weight gain than the control, whereas in high-fat fed groups, all fibre groups decreased the weight gain significantly (Table 4). Similar effects of the fibres were observed for epididymal fat pad weights, spleen and liver weights (Table 4). Blood glucose levels were not different between low- and high-fat control groups. Comparing low-fat groups, LM pectin and guar gum medium and high viscosities gave significantly lower blood glucose levels than the control group, while in high-fat fed groups, both LM and HM pectin and medium and high viscosity guar gum groups had lower glucose levels than the control group (Table 4).

**Table 4:** Weight gain (g), organ weights (g) and blood glucose levels (mmol/L) of rats (n = 7/group) fed low- or high-fat fat diets together with pectin (low-(LM) or high-methoxylated(HM)) or guar gum (low, medium or high viscosity) for three weeks.

Fat	Fibre	Weight gain	Epididymal fat	Liver	Spleen	Blood glucose
**Low**	Fibre-free	128 (14)	2.5 (0.3)	9.81 (0.8)	0.72 (0.1)	10 (1.4)
	LM Pectin	89 (13)***	1.6 (0.6)	8.2 (0.7)	0.54 (0.1)**	8.6 (0.8) *
	HM Pectin	112 (16)	2.0 (0.8)	9.6 (1.2)	0.59 (0.7)*	9.1 (0.8)
	Guar gum low viscosity	111 (14)	2.4 (0.8)	10.5 (1.5)	0.68 (0.1)	11 (1.5)
	Guar gum medium viscosity	110 (8)	1.9 (0.5)	9.3 (0.6)	0.61 (0.1)	8.7 (0.9)*
	Guar gum high viscosity	108 (7)	2.0 (0.4)	9.2 (0.6)	0.69 (0.1)	8.4 (0.6)*
**High**	Fibre-free	158 (23)	3.8 (1.0)	14.7 (3.1)	0.76 (0.1)	10 (1.1)
	LM Pectin	96 (11)***	1.4 (0.3)***	11.0 (1.2)***	0.63 (0.1)	8.3 (0.5)**
	HM Pectin	118 (6)***	2.0 (0.5)***	10.8 (0.9)***	0.61 (0.1)*	7.9 (0.4) ***
	Guar gum low viscosity	131 (7)**	2.7 (0.4)**	12.4 (0.5)*	0.71 (0.1)	9.1 (0.5)
	Guar gum medium viscosity	130 (12)**	2.3 (0.5) ***	12.4 (0.7)*	0.70 (0.1)	8.7 (0.7)*
	Guar gum high viscosity	129 (12)***	2.0 (0.4)***	12.1 (1.0)**	0.71 (0.1)	8.5 (0.5)*

Values are expressed as mean (SD).

The fibre-free control groups (low- and high-fat diet) were compared with the corresponding fibre groups and significantly different means are shown as:

* *P*-value < 0.05,

** *P*-value < 0.01 and

*** *P*-value < 0.001.

### Lipid metabolism and liver steatosis

In the blood, cholesterol levels were increased by high-fat feeding (*P*<0.001, Table 5). All guar gum groups significantly reduced cholesterol levels in the high-fat groups, while pectin groups did not. Blood triglyceride levels were significantly lower in LM-pectin and guar gum low and medium viscosities, as compared to the high-fat control (Table 5).

**Table 5:** Lipid metabolism in plasma and liver of rats (n = 7/group) fed low- or high-fat diet together with pectin (low- (LM) or high-methoxylated (HM)) or guar gum (low, medium or high viscosity) for three weeks.

Fat	Fibre	Plasma cholesterol (mg/ml)	Plasma TG (mg/ml)	Liver fat (g)	Liver cholesterol (mg/g)	Liver TG (mg/g)
**Low**	Fibre-free	1.7 (0.2)	2.2 (0.4)	0.4 (0.03)	52 (4.7)	73 (13)
	LM Pectin	1.6 (0.3)	2.0 (0.5)	0.3 (0.6)	48 (7.2)	61 (5.9)
	HM Pectin	1.4 (0.2)	1.8 (0.3)	0.3 (0.1)	49 (5.8)	61 (3.3)
	Guar gum low viscosity	1.7 (0.5)	1.5 (0.1)	0.4 (0.04)	45 (5.3)	67 (14)
	Guar gum medium viscosity	1.4 (0.3)	1.7 (0.2)	0.3 (0.03)	45 (6.4)	64 (8.2)
	Guar gum high viscosity	1.4 (0.5)	1.6 (0.1)	0.3 (0.02)	46 (1.8)	76 (34)
**High**	Fibre-free	2.7 (0.5)	2.9 (0.9)	2.4 (0.6)	234 (53)	194 (31)
	LM Pectin	2.4 (0.4)	1.9 (0.3)**	1.4 (0.4)***	182 (26)	102 (10)***
	HM Pectin	2.3 (0.4)	2.5 (0.6)	1.0 (0.1)***	136 (41)***	100 (18)***
	Guar gum low viscosity	2.0 (0.2)**	1.7 (0.2)***	2.2 (0.5)	197 (24)	212 (77)
	Guar gum medium viscosity	2.0 (0.3)**	2.1 (0.2)*	1.4 (0.3)***	149 (37)***	120 (36)**
	Guar gum high viscosity	2.2 (0.5)*	2.6 (0.4)	1.2 (0.2)***	156 (41)***	108 (36)***

Values are expressed as mean (SD).

The fibre-free control groups (low- and high-fat diet) were compared with the corresponding fibre groups and significantly different means are shown as:

* *P*-value < 0.05,

** *P*-value < 0.01 and

*** *P*-value < 0.001.

The amount of fat in the liver increased significantly with high-fat diet (*P*<0.001, Table 5). Comparing low-fat groups, none of the fibres affected liver fat content. In contrast, all fibres except guar gum low viscosity reduced liver fat significantly as compared to the high-fat control. The same results were obtained for liver triglycerides. Liver cholesterol increased with high-fat diets (*P*<0.001), and only HM pectin and guar gum medium and high viscosity groups reduced this increase in high-fat fed groups (Table 5).

### SCFA profiles in blood and caecum

**Fig 2:**
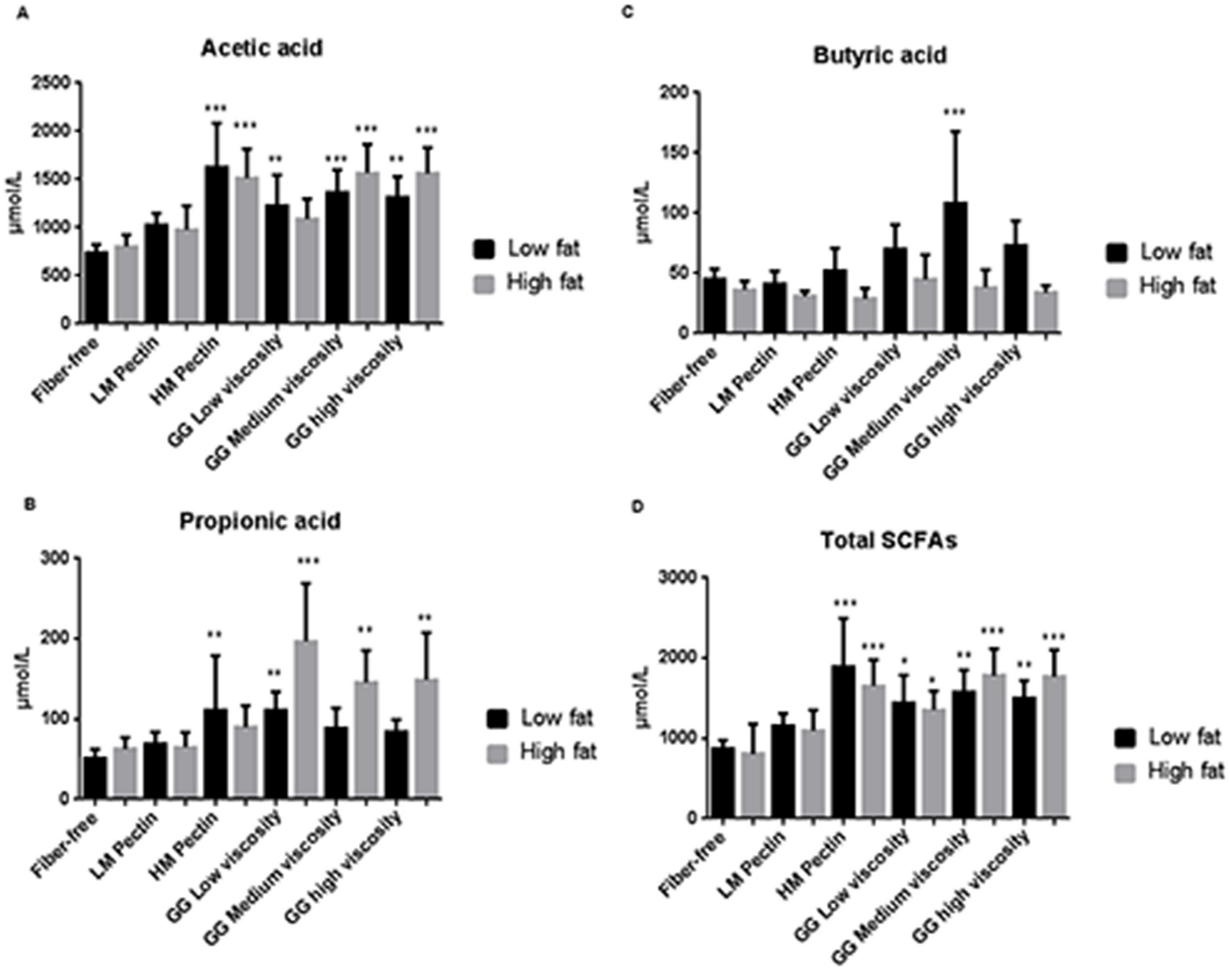
Serum levels (μmol/L) of short-chain fatty acids (SCFA) in rats (n = 7/group) fed low- or high-fat fat diets together with pectin (low- (LM) or high-methoxylated, (HM)) or guar gum (GG, low, medium or high viscosity) for three weeks. Values are expressed as mean and SD. The fibre-free control groups (low- and high-fat diet) were compared with the corresponding fibre groups and significantly different means are shown as: * *P*-value < 0.05, ** *P*-value < 0.01 and *** *P*-value < 0.001.

**Fig 3:**
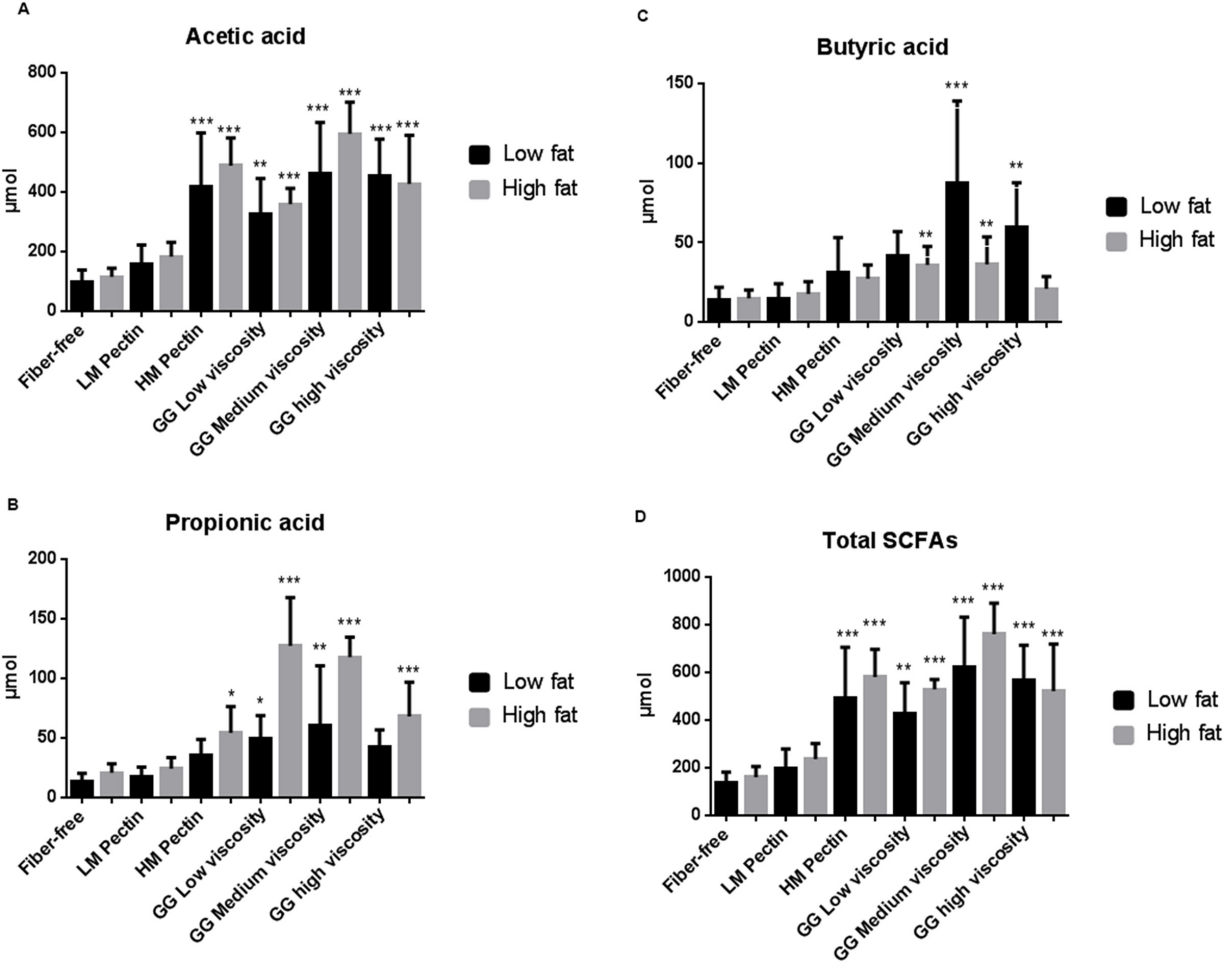
Caecal pool (μmol) of short-chain fatty acids (SCFA) in rats (n = 7/group) fed low- or high-fat fat diets together with pectin (low- (LM) or high-methoxylated (HM)) or guar gum (GG, low, medium or high viscosity) for three weeks. Values are expressed as mean and SD. The fibre-free control groups (low- and high-fat diet) were compared with the corresponding fibre groups and significantly different means are shown as: * *P*-value < 0.05, ** *P*-value < 0.01 and *** *P*-value < 0.001.

Our group has previously shown that serum levels of SCFA correlate with levels found in the caecum [25]. Overall, the total levels of serum (Fig 2) and caecal (Fig 3) SCFA and acetic acid were increased with the HM pectin and guar gum medium and high viscosity fibre diets as compared to respective low- and high-fat fibre-free control groups. In contrast, only the guar gum (low, medium and high viscosity) increased serum and caecal propionic acid in high-fat fed groups (Figs 2B and 3B). Serum butyric acid was increased in the low-fat guar gum medium viscosity group compared with the control group without any dietary fibre (Fig 2C). In the caecum, both medium and high viscosity guar gum increased butyric acid in the low-fat setting, while in high-fat groups only the medium viscosity guar gum increased butyric acid significantly (Fig 3C).

### Caecal microbiota

**Fig 4:**
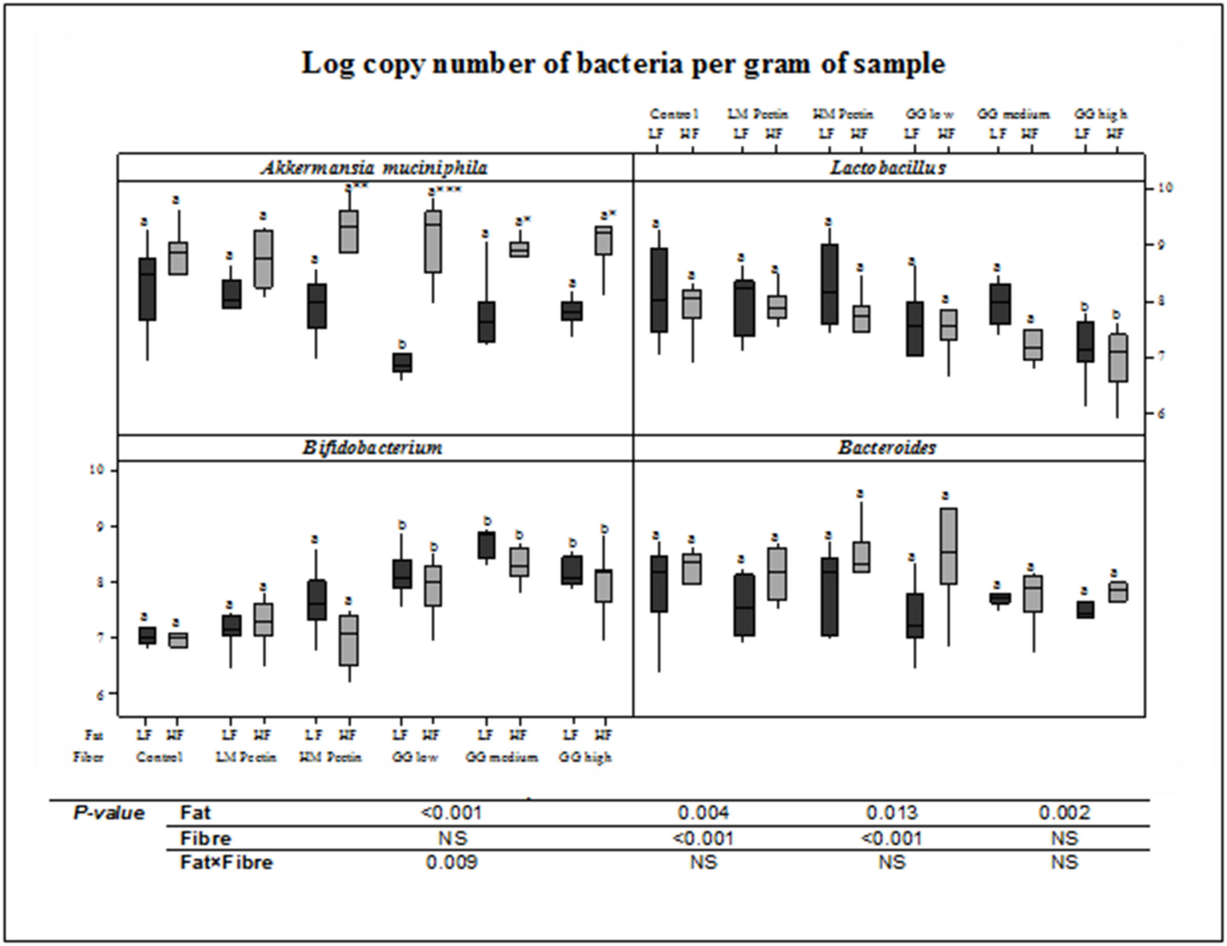
Log copy numbers of *Akkermansia muciniphila*, *Bifidobacterium*, *Lactobacillus* and *Bacteroides* per gram of caecal sample using quantitative PCR. Mean values of each fibre group (HM and LM pectin, guar gum (GG) low, medium and high viscosity) were compared to the fibre-free control, for each fat-level, and diets marked with different letters (a or b) show significant differences (*P*<0.05). Mean values of high-fat (HF) groups that were significantly different from corresponding low-fat (LF) groups were marked as follows: * *P*< 0.05, ** *P*< 0.01 and *** *P*< 0.001. Two-way analysis of variance (ANOVA) using adjusted sum of square was performed to test the interaction and main effects of each factor (fat, fibre, fat x fibre) on the caecal microbiota.

Low- and high-fat diets did not significantly differ in the caecal levels of the investigated genera *Lactobacillus*, *Akkermansia*, *Bifidobacterium* and *Bacteroides* (Fig 4). The fibre preparations did not alter *Lactobacillus* or *Bacteroides* levels, instead, they had the most effect on *Akkermansia* and *Bifidobacterium*, where both guar gum and pectin increased *Akkermansia* in the high-fat setting. *Bifidobacterium* was significantly increased by all preparations of guar gum, while pectin did not appear to have this bifidogenic effect. Two-way ANOVA of main effects and interactions between fat and fibre in the diets showed that *Akkermansia* was the most affected by the fat level in the diet, while fibre affected *Lactobacillus* and *Bifidobacterium*. In addition, the combination of high-fat and fibre addition to the diet also affected *Akkermansia* levels in caecum.

### Principal component analysis of investigated biomarkers

**Fig 5:**
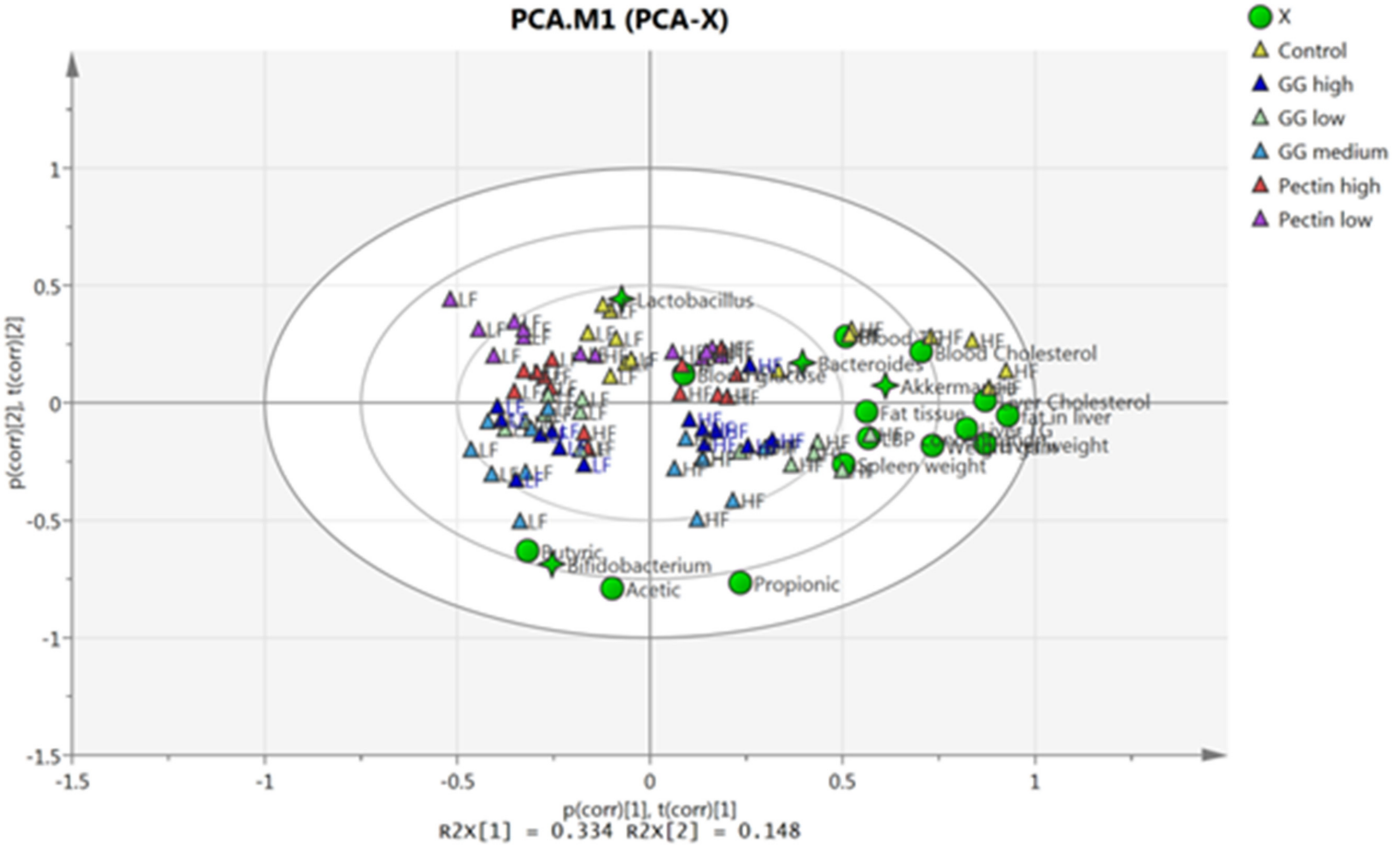
PCA biplot to visualize patterns and relationships of investigated biomarkers in rats (n = 7/group) fed low- or high-fat fat diets together with pectin (low- (LM) or high-methoxylated (HM)) or guar gum (low, medium or high viscosity) for three weeks. Triangles represent each individual rat with different colours for each fibre, LF = low fat; HF = high fat. Different biomarkers are shown in green circles and different bacterial genera in green stars.

The investigated biomarkers were included in a PCA (Fig 5). Results showed that the rats grouped according to diet, with a clear separation of low- and high-fat-fed rats. As expected, fat tissue, cholesterol and liver fat correlated with the high-fat-diet, as well as the abundance of *Akkermansia*. In contrast, *Lactobacillus* and *Bifidobacterium* were associated with low-fat diet, where *Lactobacillus* correlated with the fibre-free control group and *Bifidobacterium* with guar gum groups. In addition, butyric acid was also associated with *Bifidobacterium* (Fig 5).

## Discussion

This study was designed to elucidate how the physical and chemical properties of dietary fibres affected physiological parameters and SCFA profiles in high-fat fed rats. For this purpose, two highly fermentable fibres, pectin with two degrees of methoxylation (24% and 70%) and guar gum with low, medium and high viscosity, were used. Both fibres have a high viscosity, but guar gums are known to have a higher viscosity than pectin [26]. The fibre preparations were well tolerated by the rats, with the exception of the LM pectin. Some of the rats fed this preparation ate slightly less feed during the study, leading to transient weight loss, and we cannot exclude that this might have influenced the results.

All fibre preparations were able to reduce adiposity and body weight gain after high-fat feeding for three weeks. A study in guinea pigs fed two kinds of partially hydrolysed guar gum (i.e. hydrolysed for either 1 or 2 h) showed that both types displayed cholesterol-lowering effects [27]. In the present study, we found differences between the guar gum preparations regarding blood levels of cholesterol and TG, possibly due to the fact that we had a wider range of viscosity (from 0.02 to 600 Pa ^●^ s) than in the Santas *et al* study (ranging between 1.7 to 2.9 Pa ^●^ s) [27]. Of note, the lipid metabolism was distinctly affected by the viscosity of the guar gum, where low viscosity reduced blood cholesterol and TG levels, and high viscosity instead reduced liver steatosis. The medium viscosity guar gum incorporated characteristics of both the low and high viscosity preparations, which resulted in reduction of both blood cholesterol/TG and liver steatosis. The viscosity has been shown previously to be important for hypocholesterolaemic effects; Yamamoto *et al* (2000, [28]) showed that a high-viscosity xanthan gum—galactomannan mixture had a stronger hypolipidemic effect in rats than guar galactomannan alone, possessing a lower viscosity. Also *in vitro* work has shown that viscosity of guar gum can affect gastric lipolysis and that viscous fibres reduce lipid emulsification [29].

Interestingly, each fibre preparation gave specific SCFA profiles. In general, both pectin and guar gum increased the total levels of SCFA in both caecum and blood. Independently of viscosity, all guar gums gave high proportions of propionic acid. Regarding the methoxylation of pectin, a lower degree of methoxylation resulted in lower acetic acid in both the caecum and in the blood. A study in rats fed pectin with different degrees of methoxylation showed that pectin increased caecal SCFAs [30], which is in line with the present study. In contrast, the authors observed decreased SCFA levels with increasing degree of methoxylation, which was not observed in our experiment.

The guar gum-induced increase in total SCFA has also been shown in mice, where guar gum increased all three fatty acids, along with improved markers of the metabolic syndrome [31]. In addition, *in vitro* experiments with guar gum fermented in the presence of human fecal microbiota gave rise to higher levels of SCFA than the control [32]. In the present study, the viscosity of guar gum gave rise to distinct SCFA profiles, where low and medium viscosity increased caecal butyric acid in both low- and high-fat-fed groups. In serum, only the medium viscosity guar gum increased butyric acid, in the low-fat group. This could be related to viscosity-dependent alterations in the gut microbiota. Hence, we further analysed three bacterial genera in caecal samples: *Lactobacillus*, *Bifidobacterium* and *Bacteroides* as well as the species *Akkermansia muciniphila*. These bacteria have previously been implicated in studies with dietary fibre/prebiotics and high-fat feeding. The pectin and guar gum preparations affected the microbiota differently, which most probably was due to their different sugar monomeric compositions and type of linkages between the monomeric units. Low viscosity preparations of guar gum are likely fermented more rapidly than higher viscosity preparations, thus stimulating the growth of different types of bacteria. We found no significant difference in *Lactobacillus* levels, but the amount of *Akkermansia muciniphila* was significantly lower in the low-fat guar gum low viscosity group. This bacterium has been shown to increase with fructo-oligosaccharide feeding and *A*. *muciniphila* treatment has been claimed to reverse effects of diet-induced obesity [21]. The highest levels of *Akkermansia* were found in rats fed diets with a combination of high amounts of fat together with fibres, the reason for this interaction between fibres and fat needs further investigation. Regarding the levels of *Bifidobacterium*, all guar gum diets increased *Bifidobacterium*, but not diets with pectin, illustrating a bifidogenic effect specifically related to guar gum. The bifidogenic effect of guar gum has been demonstrated previously in humans consuming partially hydrolysed guar gum [33]. In fact, also butyrate-producing bacteria were enriched [33].

In the colon, the degradation of dietary fibre is accomplished collectively by microbial consortia, catalysed by specific enzymes [34]. For galactomannan the process is mainly catalysed by glycoside hydrolase (GH) enzymes [35]. For pectin the main enzymes involved are pectin hydrolases and lyases. The abundance and type of enzymes encoded by different species of the gut microbiota vary greatly. Pectin is known to be highly fermented in human colon [36]. In rats, low methoxylated pectin was shown to be fermented to a higher extent than high methoxylated [30] but the reason remain unclear. One explanation could be that this type of pectin can form more cross-links between the carboxyl groups of the uronic acid units than HM pectin, leading to a higher resistance to microbial fermentation. Among the bacteria that can grow on pectin and guar gum *in vitro* are certain *Bifidobacterium* as well as *Bacteroides* strains [6, 37–39]. In our study LM pectin seemed to be more or less resistant to fermentation, as judged by the low caecal amounts of SCFA with this material. However, it might be questioned if this type of pectin was resistant to fermentation or if it was due to the lower feed intake in this group of rats.

Galactomannan depolymerisation is catalysed by β-mannanase, well described for several environmental bacteria [40]. In our study, *Lactobacillus* and *Akkermansia* did not increase in abundance when the rats were fed with guar galactomannan. When examining the CAZy database of glycoside hydrolases (www.cazy.org [41]) none of the genomes of sequenced *Lactobacillus* or *Akkermansia* are predicted to harbour genes encoding β-mannanases. Interestingly, *Bifidobacterium* increased in abundance when the rats were fed with guar gum of all molecular weights (Fig 4) and several *Bifidobacterium* species are predicted to harbour β-mannanases. Furthermore, recently a β-mannanase from *B*. *adolescentis* was characterized in detail and was found to hydrolyse guar galactomannan [42]. In *Bacteroides ovatus* a β-mannanase was shown to be induced in the presence of guar gum [43]. Furthermore, *B*. *ovatus* has both gene sets for degradation of galactomannan and resistant pectin polysaccharides, but for example the closely related *Bacteroides thetaiotaomicron* has gene set to grow on pectin, but not on galactomannan [38]. However, in the current study, the number of *Bacteroides spp*. did not vary significantly in the presence or absence of guar gum or pectin. Nevertheless, it must be kept in mind that the colon is a complex system, containing an enormous number of bacteria excreting various enzymes with different activities, and single biochemical reactions may not be reflecting the entire enzymatic activity of the colonic microbiota. Our microbiota analyses were limited to three bacterial genera and one species commonly associated with fibre intake, and future work would benefit from performing complete 16S rRNA gene sequencing, which would provide a more comprehensive view on the impact of guar gum and pectin on the rats’ caecal microbiota.

In the present study, we used twelve diets to investigate how the physical and chemical properties of guar gum and pectin affected metabolic responses in rats fed low- and high-fat diets. Regarding viscosity of guar gum, we surprisingly found that the medium viscosity was the most optimal one for achieving lipid-lowering effects and for increasing butyric acid in the caecum and blood. Butyric acid was correlated with bifidobacterial numbers. However, bifidobacteria are not able to produce butyrate, instead, they likely provide partial degradation products of fibres that other bacteria can utilize for butyrate production [44].

## Conclusions

The degree of methoxylation and viscosity of dietary fibres was of importance for metabolic effects and gave rise to distinct SCFA profiles as well as specific alterations in the gut microbiota. The medium viscosity guar gum was the most effective preparation for prevention of diet-induced hyperlipidaemia and liver steatosis. The caecal abundance of *Bifidobacterium* was the highest in rats fed the medium viscosity guar gum, which was also associated with butyrate levels, both in caecum and in the systemic circulation. Which bacterial species that are involved in guar galactomannan utilization is presently unknown and need further investigation. Taken together, by tailoring the viscosity and possibly also the methoxylation of dietary fibres, metabolic effects may be optimized, through a targeted modulation of the gut microbiota and its metabolites.

## Supporting Information

S1 Fig Body weight in rats (n = 7/group) fed low- (A) or high-fat (B) diets together with pectin (low- (LM) or high-methoxylated, (HM)) or guar gum (GG, low, medium or high viscosity) for three weeks. Values are expressed as mean and SD.
(TIF)
