# Supplementary figures and images for: The Physico-Chemical Properties of Dietary Fibre Determine Metabolic Responses, Short-Chain Fatty Acid Profiles and Gut Microbiota Composition in Rats Fed Low- and High-Fat Diets
Source: PLoS One. 2015 May 14;10(5):e0127252. doi: 10.1371/journal.pone.0127252 (PMC4431822; doi:10.1371/journal.pone.0127252)

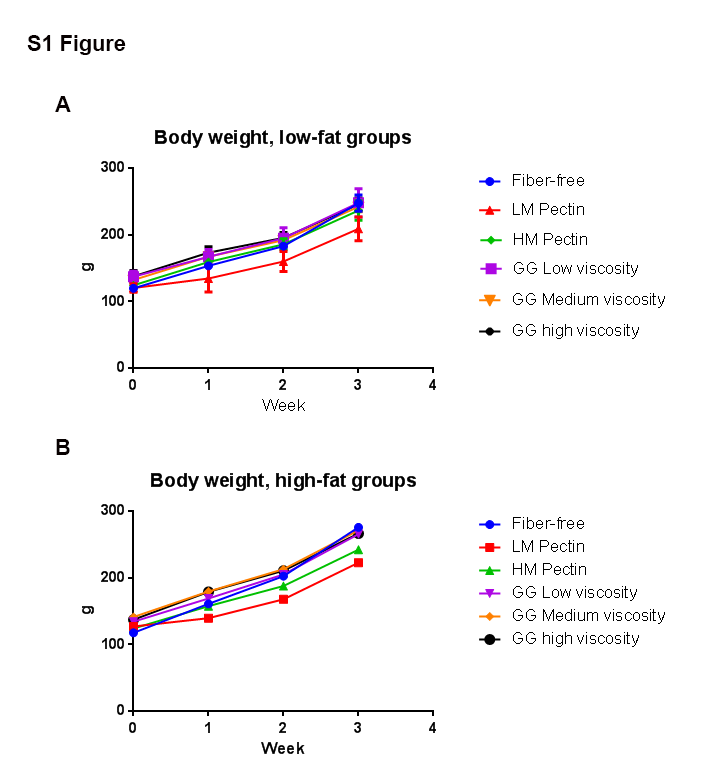

Supplement: S1 Fig — Values are expressed as mean and SD. (TIF) [file pone.0127252.s001.tif]
